# Long-term outcomes and age-dependent recovery trajectories following short-term spinal cord stimulation in patients with disorders of consciousness

**DOI:** 10.3389/fneur.2026.1886072

**Published:** 2026-08-14

**Authors:** Yuye Liu, Chen He, Xiaoli Geng, Xueling Chen, Jianghong He

**Affiliations:** 1 Beijing Neurosurgical Institute, Beijing, China; 2 Department of Neurosurgery, Beijing Tiantan Hospital, Capital Medical University, Beijing, China

**Keywords:** long-term follow up, PDOC, prolonged disorders of consciousness, spinal cord stimulation, stSCS

## Abstract

**Objective:**

To evaluate long-term changes in consciousness and age-related recovery patterns in patients with prolonged disorders of consciousness (pDoC) following spinal cord stimulation (stSCS), and to explore whether early postoperative neurological improvement is associated with long-term outcomes.

**Methods:**

This retrospective study included 30 patients with pDoC who underwent stSCS and were followed for 24 months. Consciousness was assessed using the Coma Recovery Scale–Revised (CRS-R) at baseline, 3 days, 14 days, 12 months, and 24 months. Patients were stratified into younger (≤50 years) and older (>50 years) groups. Longitudinal changes in CRS-R scores were analyzed using linear mixed-effects models, and linear regression was performed to evaluate the association between early neurological improvement at 14 days and 12-month outcomes.

**Results:**

CRS-R scores progressively increased during the 24-month follow-up, with significantly higher scores at 12 months (10.47 ± 6.52) and 24 months (11.84 ± 6.38) compared with baseline (6.17 ± 2.67, both *p* < 0.001). Analysis of CRS-R changes from baseline suggested different recovery patterns between age groups. Younger patients showed earlier within-group improvement, although overall recovery trajectories and long-term outcomes were comparable between age groups. However, no significant differences in overall CRS-R improvement were observed between the two groups. The distribution of clinical consciousness states was also comparable between groups during follow-up. Early improvement at 14 days was significantly associated with 12-month CRS-R scores in both the Younger Group (R^2^ = 0.536, *p* = 0.0019) and the Older Group (R^2^ = 0.810, *p* < 0.0001).

**Conclusion:**

Patients with pDoC showed sustained CRS-R improvement after stSCS during long-term follow-up. Age appeared to influence the pattern of recovery, with younger patients showing earlier improvement, although overall long-term outcomes were comparable between age groups. Early improvement was associated with better long-term outcomes, suggesting potential prognostic value. The specific contribution of stSCS requires further validation in prospective controlled studies.

## 1 Introduction

Severe brain injury leading to prolonged disorders of consciousness (pDoC) remains a major challenge in neurosurgery and rehabilitative medicine (1). Patients in an unresponsive wakefulness syndrome (UWS), also known as vegetative state, or a minimally conscious state (MCS) require long-term care, which imposes a substantial socioeconomic burden on families and healthcare systems (2, 3). While traditional pharmacological and sensory stimulation therapies offer limited benefits for many patients, neuromodulation techniques have emerged as potential interventions to facilitate consciousness recovery (4–7). Importantly, these neuromodulatory strategies are intended to complement rather than replace conventional rehabilitation, acting as neural primers that elevate cortical excitability and render the brain more receptive to standard physical and sensory inputs.

Spinal cord stimulation (SCS) has been increasingly utilized to promote arousal in patients with pDoC by modulating the ascending reticular activating system and increasing cerebral blood flow (5). Among the various modalities, short-term spinal cord stimulation (stSCS) has gained traction in clinical practice (8–10). Unlike permanent SCS, which requires surgical implantation of an internal pulse generator, stSCS involves the temporary percutaneous placement of epidural electrodes connected to an external stimulator for a defined duration, typically two to four weeks. This approach is often preferred due to its lower risk of long-term implant-related complications (11, 12), such as infection, lead migration, or hardware failure, while offering high flexibility in parameter adjustment during the stimulation phase. Furthermore, stSCS is increasingly recognized not just as a temporary screening tool, but as a transient physiological catalyst capable of triggering enduring endogenous neuroplasticity (13, 14). However, the current literature primarily focuses on short-term or mid-term outcomes, typically ranging from several weeks. There is a lack of evidence regarding the long-term outcomes and functional stability of stSCS over an extended follow-up period.

Patient age is another critical factor that influences neuroplasticity and clinical prognosis following brain injury (15–17). In many neurosurgical contexts, advanced age is associated with reduced physiological reserve and a higher incidence of comorbidities, which may limit the potential for neurological recovery (17). Nevertheless, it remains unclear whether age-related differences significantly affect the ultimate therapeutic ceiling of stSCS or the temporal pattern of consciousness emergence. Establishing whether older patients can achieve comparable long-term gains to their younger counterparts is essential for the equitable application of neuromodulation therapies.

Furthermore, the high cost and intensive nature of pDoC rehabilitation necessitate the early identification of patients with high recovery potential. The 14-day post-intervention mark, which coincides with the completion of the stSCS stimulation phase, represents a potential window for prognostic assessment. Identifying a correlation between early clinical responses and long-term functional outcomes would assist clinicians in managing family expectations and optimizing individualized rehabilitation protocols.

This study aims to evaluate the clinical outcomes of stSCS as an adjunctive therapy combined with conventional rehabilitation therapies in patients with pDoC over a 24-month follow-up period. We specifically compare longitudinal clinical outcomes between younger and older patient cohorts to explore age-related differences in the timing and magnitude of improvement. Additionally, we analyze the relationship between the clinical response at 14 days and the long-term Coma Recovery Scale-Revised (CRS-R) scores to evaluate the prognostic value of early neurofunctional changes.

## 2 Materials and methods

### 2.1 Study design and participants

This single-center retrospective cohort study was conducted to evaluate the long-term follow-up clinical outcomes of stSCS in patients, and compare the application of different age strategies. We consecutively enrolled pDoC patients who were admitted to the Department of Neurosurgery, Beijing Tiantan Hospital, Capital Medical University, and underwent stSCS surgery between May and August 2023. The study protocol was reviewed and approved by the Ethics Committee of Beijing Tiantan Hospital (Approval No. KY2023-175-03). As the participants were unable to provide informed consent due to their clinical status, written informed consent was obtained from their immediate family members or legal guardians.

Inclusion criteria were as follows: (1) met the clinical diagnostic criteria for pDoC, including UWS and MCS (5); (2) underwent stSCS treatment during the subacute stage, with a disease onset exceeding 28 days; (3) written informed consent for both the surgery and the study was signed by legal guardians.

Patients were excluded if they met any of the following criteria: (1) progressive intracranial lesions, such as uncontrolled brain tumors or active hydrocephalus requiring imminent surgical shunt adjustment, which would confound the evaluation of consciousness recovery; (2) severe spine deformities or local infections at the implantation site that technically precluded safe spinal cord electrode placement; or (3) pre-existing, severe, irreversible organic organ failure prior to stSCS that was expected to cause poor short-term survival independent of neurological status. Notably, patients who died of systemic clinical complications during the 24-month follow-up were not excluded from the baseline cohort to avoid survival bias and ensure a realistic representation of long-term prognosis.

Patients enrolled in this study were dichotomized into the Younger Group (≤50 years old) and the Older Group (>50 years old). This age threshold was selected based on previous studies suggesting that aging may be associated with progressive alterations in brain structural and functional plasticity, which could potentially influence neurological recovery (18, 19). Furthermore, this classification aligned with the stratification protocols used in recent large-scale prospective cohort studies on prolonged pDoC (20), while simultaneously ensuring a statistically balanced distribution of sample sizes between the two comparison groups in this study.

### 2.2 Surgical procedures

All surgeries were performed by the same experienced team of neurosurgeons and anesthesiologists. Once stable anesthesia was achieved, the patient was placed in the prone position. Under C-arm fluoroscopic guidance, percutaneous puncture was performed at the T7/8 interspace. An 8-contact electrode (Model 3,777, Medtronic, USA) was precisely inserted into the spinal epidural space and advanced cranially to the C2 vertebral level. After confirming the electrode position via multi-angle fluoroscopy, the lead was connected to an external temporary stimulator (Model 37,022, Medtronic). The core technical details of the surgery followed the standardized protocols previously described by our team (4, 10).

### 2.3 Postoperative stimulation protocol

All patients underwent cervical CT scan with 3D reconstruction within 12 h post-surgery. The primary objectives were to confirm the precise placement of the electrode tip in the posterior epidural space at the C2 vertebral level and to exclude early technical complications such as spinal cord injury, epidural hematoma, or electrode displacement (4).

To avoid the influence of the acute post-traumatic phase, stSCS commenced 1–2 days after surgery once vital signs were stable. Stimulation was delivered at a frequency of either 5 or 70 Hz with a pulse width of 210 μs, using individualized sub-sensory threshold voltages. Parameter selection was performed based on each patient’s neurophysiological response during a standardized pre-treatment EEG assessment phase (4). The selected parameters were subsequently maintained throughout the 14-day stimulation period. In total, 13 patients received 5-Hz stimulation and 17 patients received 70-Hz stimulation. Intermittent stimulation was administered for approximately 12 h daily during daytime hours, following a cycle of 15 min on and 15 min off. This stimulation cycle continued for 14 days, after which the temporary electrode was removed under sterile conditions in the ward.

### 2.4 Clinical outcomes and data collection

We recorded the patients’ baseline medical information from the electronic medical record system, with the primary outcome measure being the change in CRS-R scores. To overcome geographical barriers and ensure safety during long-term follow-up, a standardized video-assisted CRS-R protocol was implemented. The recordings were obtained using a standardized protocol designed to capture the behavioral responses required for CRS-R scoring. All videos were independently reviewed by two senior neurosurgery who were blinded to the patients’ age-group allocation and previous assessment results. In cases of disagreement, a third experienced neurologist was consulted to reach consensus.

Assessment time points included: baseline (the highest score from at least three assessments conducted upon clinical stability after admission), stimulation day 3, day 14 (immediately prior to electrode removal), together with a long-term follow-up at 12-month and 24-month post-surgery. All assessments were performed by two clinical physicians who had undergone consistency training.

### 2.5 Statistical analysis

Statistical analysis was performed using IBM SPSS Statistics version 26.0 and GraphPad Prism 9.0. Continuous variables were first assessed for normality using the Shapiro–Wilk test. Normally distributed data were expressed as mean with standard deviation, and intergroup comparisons were conducted using the independent samples t test. Non-normally distributed data were presented as median with interquartile range, with intergroup comparisons performed using the Mann–Whitney U test. Categorical variables were compared between groups using the chi-square test or Fisher exact test.

To avoid assigning arbitrary postmortem CRS-R scores, deceased cases were reported separately and their subsequent observations were treated as missing. Longitudinal changes in CRS-R scores were analyzed using a linear mixed-effects model (REML estimation), with follow-up time treated as a fixed effect and subject as a random effect to account for repeated measurements and missing observations caused by death during follow-up. Pairwise comparisons between time points were adjusted using Tukey’s multiple comparisons test. For age-stratified analyses, age group (Younger vs. Older), follow-up time, and their interaction were included as fixed effects, while subject was treated as a random effect. *Post hoc* comparisons were corrected using Šídák’s multiple comparisons test. Continuous variables are presented as mean ± standard deviation (SD). Statistical significance was defined as *p* < 0.05. Separate linear regression analyses were performed within each age group, using the 14-day ΔCRS-R as the independent variable and the absolute 12-month CRS-R score as the dependent variable.

## 3 Results

### 3.1 Baseline characteristics

Thirty patients were enrolled in the study and completed baseline evaluation. The mean ages for the two groups were 29.40 ± 10.13 years and 57.80 ± 4.90 years, respectively, showing a statistically significant difference (*p* < 0.001). Aside from age, no significant differences were observed between the two groups across other baseline clinical variables. Specifically, the distribution of gender (*p* = 0.699), etiology (*p* = 0.537), and clinical diagnosis (*p* = 0.715) remained comparable. Furthermore, there were no statistically significant disparities in disease duration (*p* = 0.576) or preoperative CRS-R scores (*p* = 0.739). These results indicate that the baseline characteristics, other than age, were well-balanced between the two cohorts, providing a solid foundation for subsequent comparative analysis.

**Figure 1:**
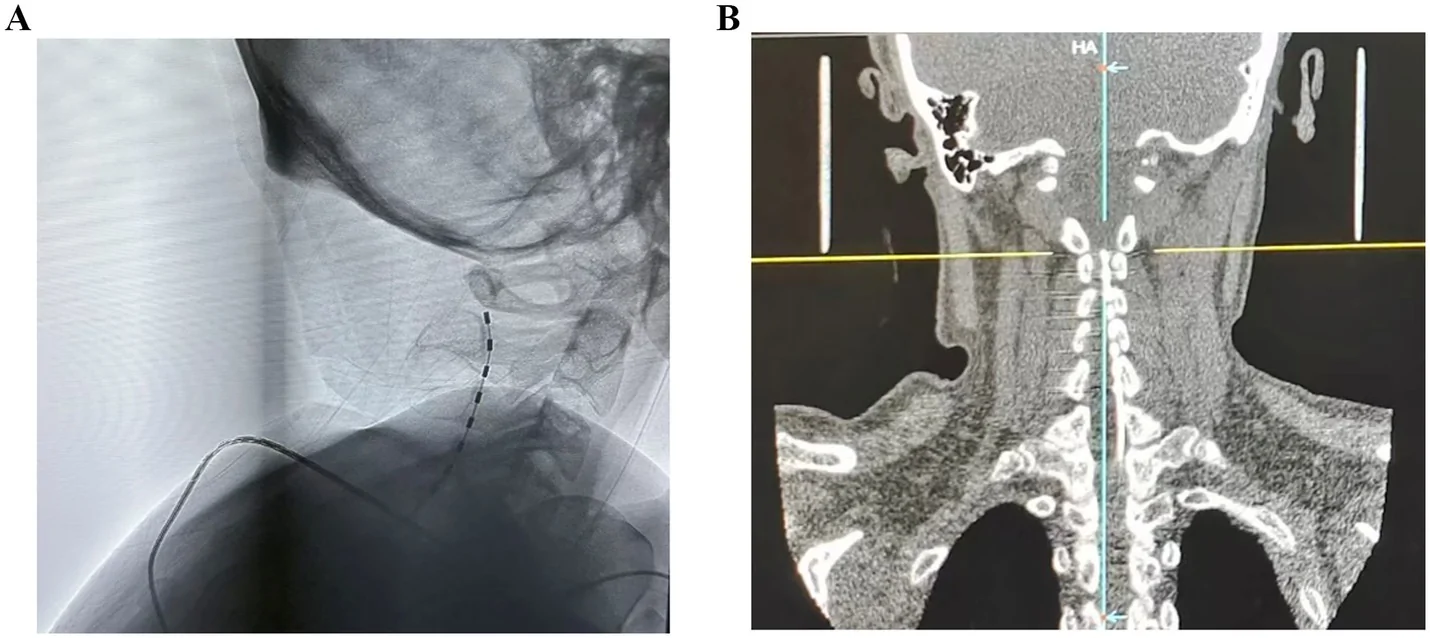
**(A)** Intraoperative lateral digital radiography image. **(B)** Postoperative coronal t CT image of the cervical spine (tissue window).

All 30 patients underwent successful percutaneous lead implantation without technical failure. Intraoperative fluoroscopy demonstrated the smooth advancement of the 8-contact electrodes, with the terminal ends reaching the C2 segment for real-time localization (Figure 1A). Postoperative assessments validated the electrode alignment, showing no displacement from the intended path (Figure 1B). Specifically, the leads were correctly situated in the posterior epidural space behind C2. This concordance between intraoperative and postoperative findings confirmed the absence of early perioperative complications and the accuracy of the implantation technique.

### 3.2 Longitudinal changes in CRS-R scores

**Figure 2:**
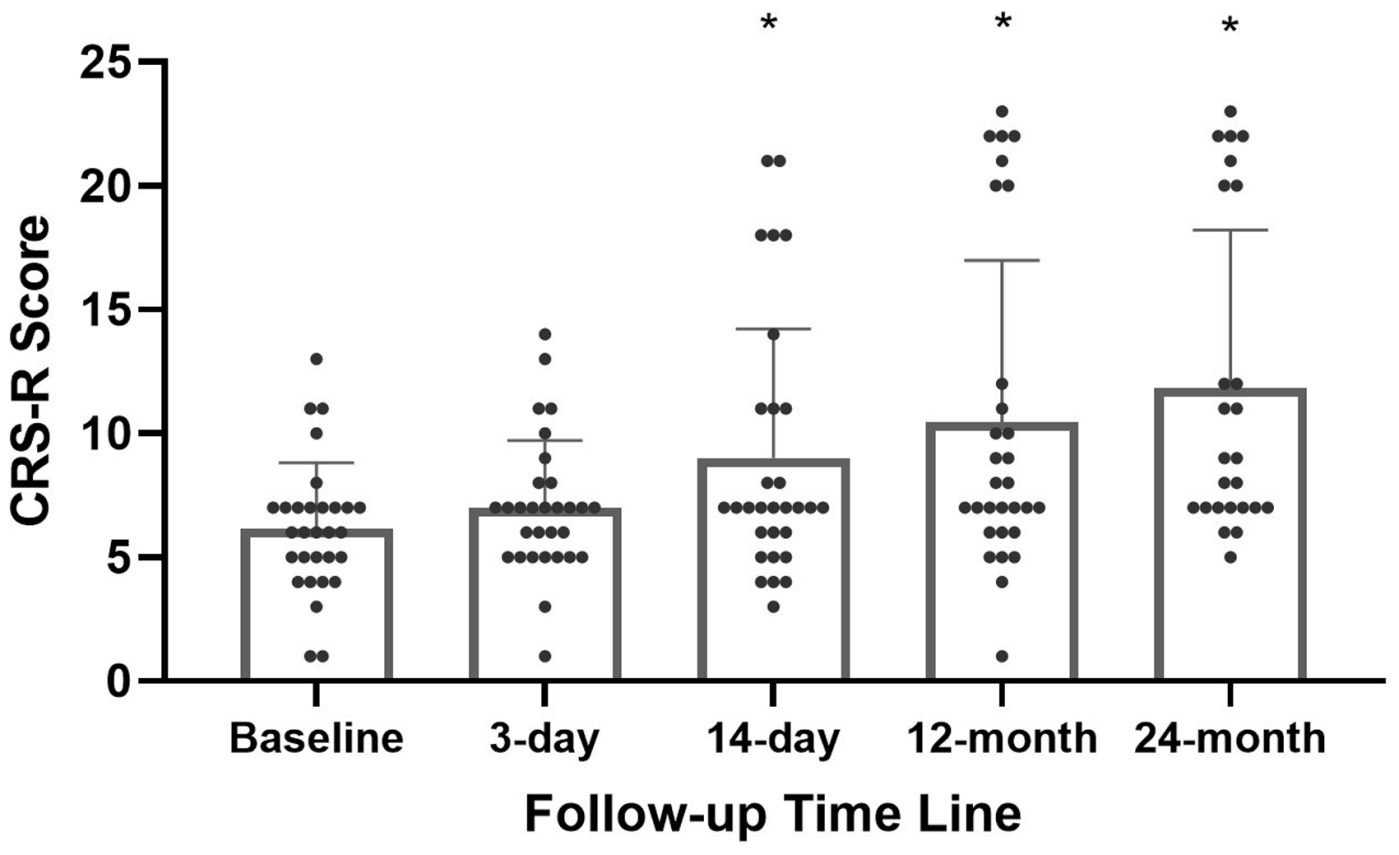
Changes in CRS-R scores for the entire cohort during follow-up. Bars represent mean ± SD, and dots indicate individual patients. Statistical significance was assessed using a linear mixed-effects model with Tukey’s multiple comparisons test. *p < 0.05 versus baseline.

During follow-up, two patients in the Younger group and three patients in the Older group died before the 24-month assessment; therefore, CRS-R scores at 24 months were available for 25 patients. The dynamic changes in CRS-R scores across the different follow-up time points are illustrated in Figure 2. Longitudinal analysis using a linear mixed-effects model demonstrated a significant effect of follow-up time on CRS-R scores (*p* < 0.0001), indicating continuous neurological improvement after stSCS treatment. The mean CRS-R score increased from 6.17 ± 2.67 at baseline to 7.00 ± 2.73 at 3 days, 9.00 ± 5.23 at 14 days, 10.47 ± 6.52 at 12 months, and 11.84 ± 6.38 at 24 months. Compared with baseline, CRS-R scores were significantly increased at 14 days (*p* = 0.008), 12 months (*p* < 0.0001), and 24 months (p < 0.0001), whereas the increase observed at 3 days did not reach statistical significance. The scatter distribution further demonstrated considerable inter-individual variability, although the majority of patients exhibited sustained neurological improvement throughout follow-up.

### 3.3 Age-related neurological recovery after stSCS

**Figure 3:**
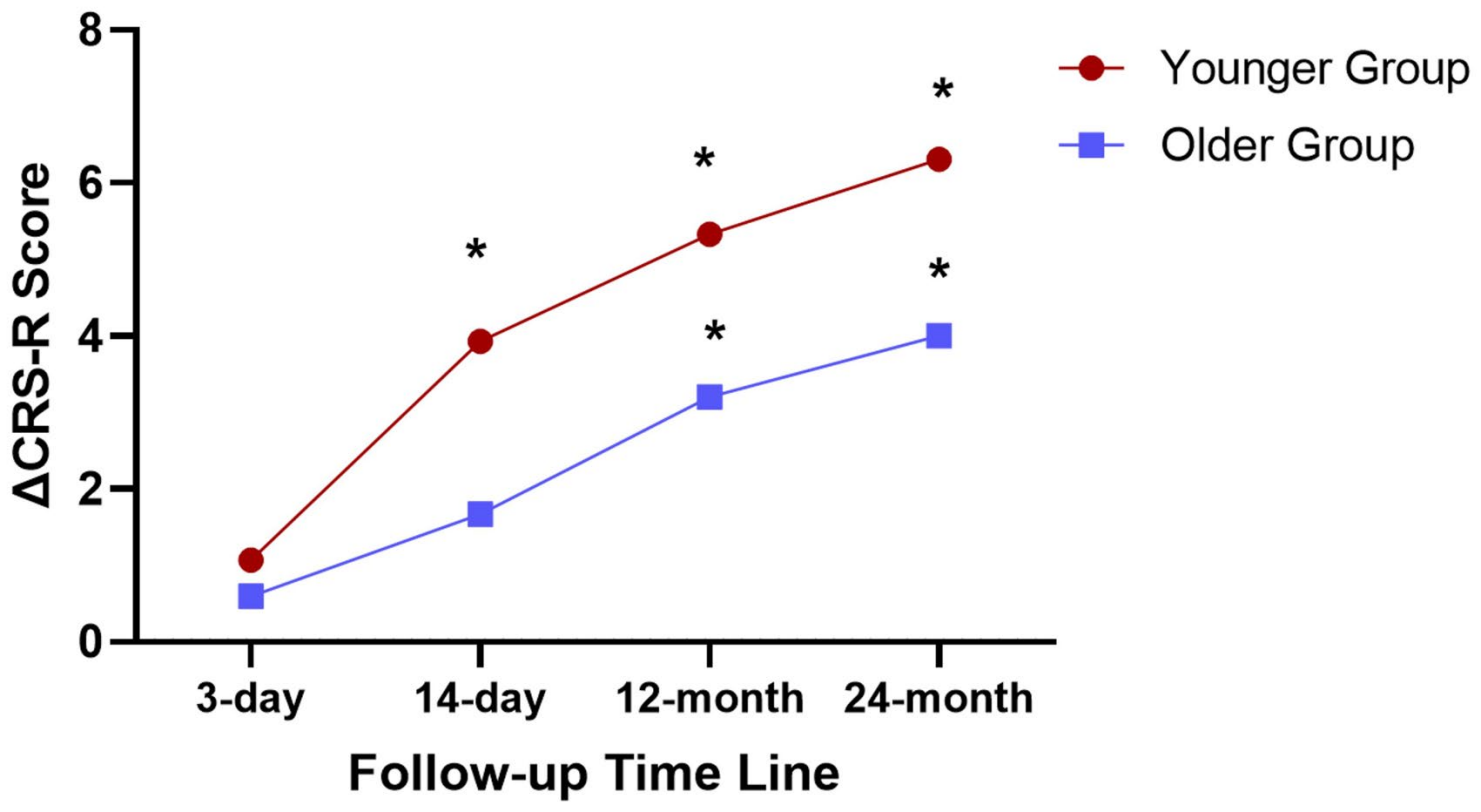
Age-related changes in neurological improvement following stSCS. Longitudinal changes in CRS-R improvement from baseline (ΔCRS-R) in the Younger and Older groups following stSCS.ΔCRS-R was calculated as the difference between each follow-up CRS-R score and the corresponding baseline score. Asterisks indicate significant differences compared with baseline within each age group after multiple-comparison correction (*p < 0.05). Between-group differences at individual follow-up time points were not statistically significant.

Longitudinal changes in CRS-R improvement from baseline (ΔCRS-R) were analyzed according to age group (Figure 3). Baseline CRS-R scores were comparable between the Younger Group and Older Group (6.00 ± 1.77 vs. 6.33 ± 3.39, *p* = 0.739). Both groups demonstrated progressive increases in ΔCRS-R during follow-up after stSCS. Linear mixed-effects analysis revealed a significant effect of follow-up time on ΔCRS-R (*p* = 0.0008), whereas neither the main effect of age group (*p* = 0.1312) nor the age group-by-time interaction (*p* = 0.4641) reached statistical significance. No significant differences in ΔCRS-R values were observed between the two groups at individual follow-up time points after correction for multiple comparisons.

Although the overall magnitude of improvement was comparable, subtle differences in the timing of clinical responses were observed between groups. Compared with baseline, the Younger Group showed significant increases in absolute CRS-R scores at 14 days (9.93 ± 5.13, *p* = 0.010), followed by sustained improvement at 12 months (11.33 ± 6.47, *p* = 0.0002) and 24 months (12.23 ± 6.55, *p* = 0.002). In contrast, the Older Group demonstrated a more gradual recovery pattern, with significant improvements observed at 12 months (9.60 ± 6.69, *p* = 0.048) and 24 months (11.42 ± 5.42, *p* = 0.009), whereas the improvement at 14 days did not reach statistical significance (8.07 ± 5.33, *p* = 0.563). These findings suggest that younger patients may exhibit earlier responsiveness after stSCS, whereas older patients may require a longer period to demonstrate measurable clinical improvement (Table 1).

**Table 1:** Comparison of baseline characteristics.

Characteristics	Total (*n* = 30)	Younger group (*n* = 15)	Older group (*n* = 15)	*P*
Gender				0.699
Male	20	11	9	
Female	10	4	6	
Age	42.17 ± 16.46	29.40 ± 10.13	57.80 ± 4.90	<0.001
Etiology				0.537
Ischemia and hypoxia	8	5	3	
Trauma	7	4	3	
Stroke	15	6	9	
Disease duration (months)	4 [2, 6]	5 [2, 6]	4 [2, 6]	0.576
Preoperative CRS-R score	6.17 ± 2.67	6.00 ± 1.77	6.33 ± 3.39	0.739
Clinical diagnosis				0.715
MCS	14	6	8	
UWS	16	9	7	

CRS-R, the Coma Recovery Scale-Revised; MCS, minimally conscious state; UWS, unresponsive wakefulness syndrome.

### 3.4 Recovery of CRS-R subscales

**Figure 4:**
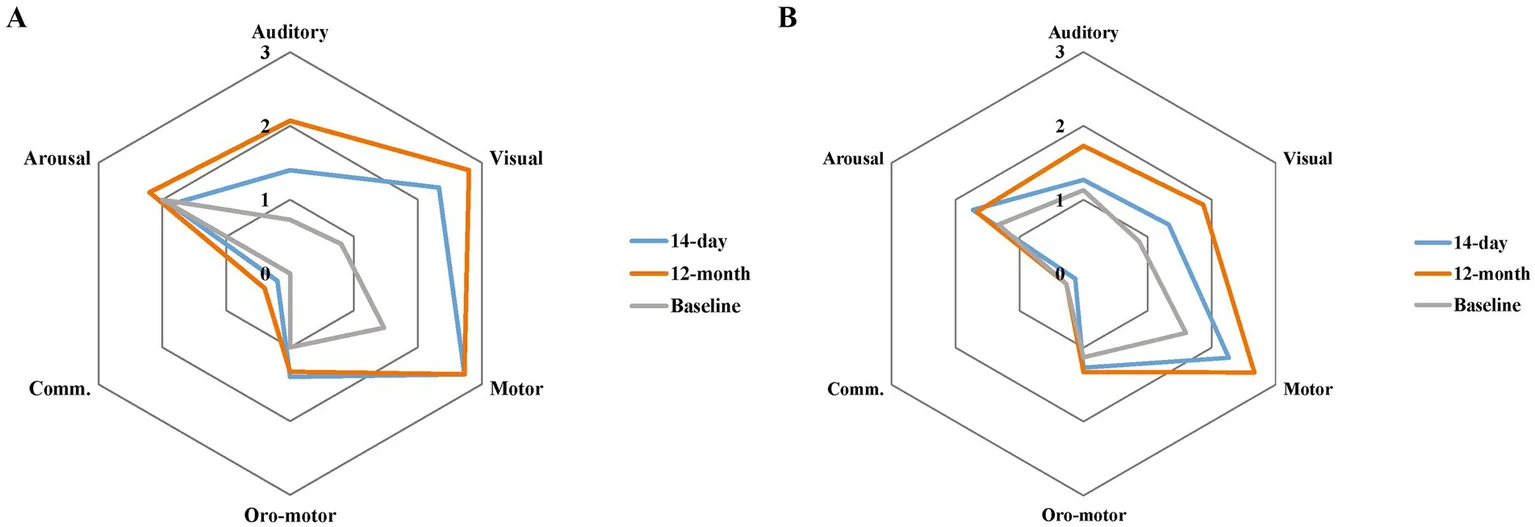
Multidimensional recovery profiles and longitudinal evolution of CRS-R subscale scores. **(A)** Younger Group and **(B)** Older Group. Data represent mean scores across six functional domains at baseline, 14 days, and 12 months.

The longitudinal changes in CRS-R subscale scores are presented in Figure 4A (Younger Group) and Figure 4B (Older Group). At baseline, both groups exhibited severe impairments across all CRS-R domains. During follow-up, improvements were observed primarily in the visual, motor, and auditory subscales, whereas communication and oromotor/verbal function showed relatively limited recovery in both groups. Figure 4 illustrates the longitudinal recovery pattern across CRS-R subscales rather than statistical comparisons between subscales.

In the Younger Group, recovery became evident as early as 14 days after surgery, with notable increases in the visual subscale (from 0.80 to 2.33) and motor subscale (from 1.47 to 2.73). During long-term follow-up, auditory function continued to improve, increasing from 1.40 at baseline to 2.07 at 12 months, while motor function remained relatively stable after the initial postoperative improvement. In contrast, communication and oromotor/verbal scores remained low throughout the follow-up period.

The Older Group demonstrated a more gradual pattern of recovery. Early improvements were also observed in the visual and motor domains, although the magnitude of change appeared smaller than that in the Younger Group. Greater inter-individual variability was observed during long-term follow-up, reflected by the wider dispersion of CRS-R total scores at 12 months (SD = 6.69).

Overall, recovery following stSCS was characterized by greater improvements in sensory and motor-related domains than in higher-order communication functions, with younger patients showing a trend toward descriptively earlier functional gains.

### 3.5 Evolution of clinical consciousness states during follow-up

Beyond numerical score improvements, the qualitative transition of consciousness states further characterizes the recovery trajectory for both cohorts (Table 2). As early as 14 days after surgery, three patients (20.0%) in the Younger Group and two patients (13.3%) in the Older Group had transitioned to emergence from minimally conscious state (EMCS). By 12 months, ten patients (five in each group) had reached EMCS. At the 24-month follow-up, all deaths occurred in patients who had been classified as UWS at baseline (two in the Younger Group and three in the Older Group). Among the remaining patients, recovery was generally sustained or continued to improve over time. Although the Younger Group, despite having a higher proportion of baseline UWS cases, appeared to show a more rapid early decline in UWS, the overall distribution of consciousness states remained comparable between age groups throughout follow-up (*p* = 0.986).

**Table 2:** Distribution of consciousness states in follow-up.

Groups	Follow-up Interval	EMCS	MCS	UWS	Deceased
Younger group	Baseline	0	6	9	0
	14-day	3	10	2	0
	12-month	5	7	3	0
	24-month	4	8	1	2
Older group	Baseline	0	8	7	0
	14-day	2	5	8	0
	12-month	5	6	4	0
	24-month	5	5	2	3

EMCS, Emergence from minimally conscious state; MCS, minimally conscious state; UWS, unresponsive wakefulness syndrome.

### 3.6 Association between early improvement and long-term outcome

**Figure 5:**
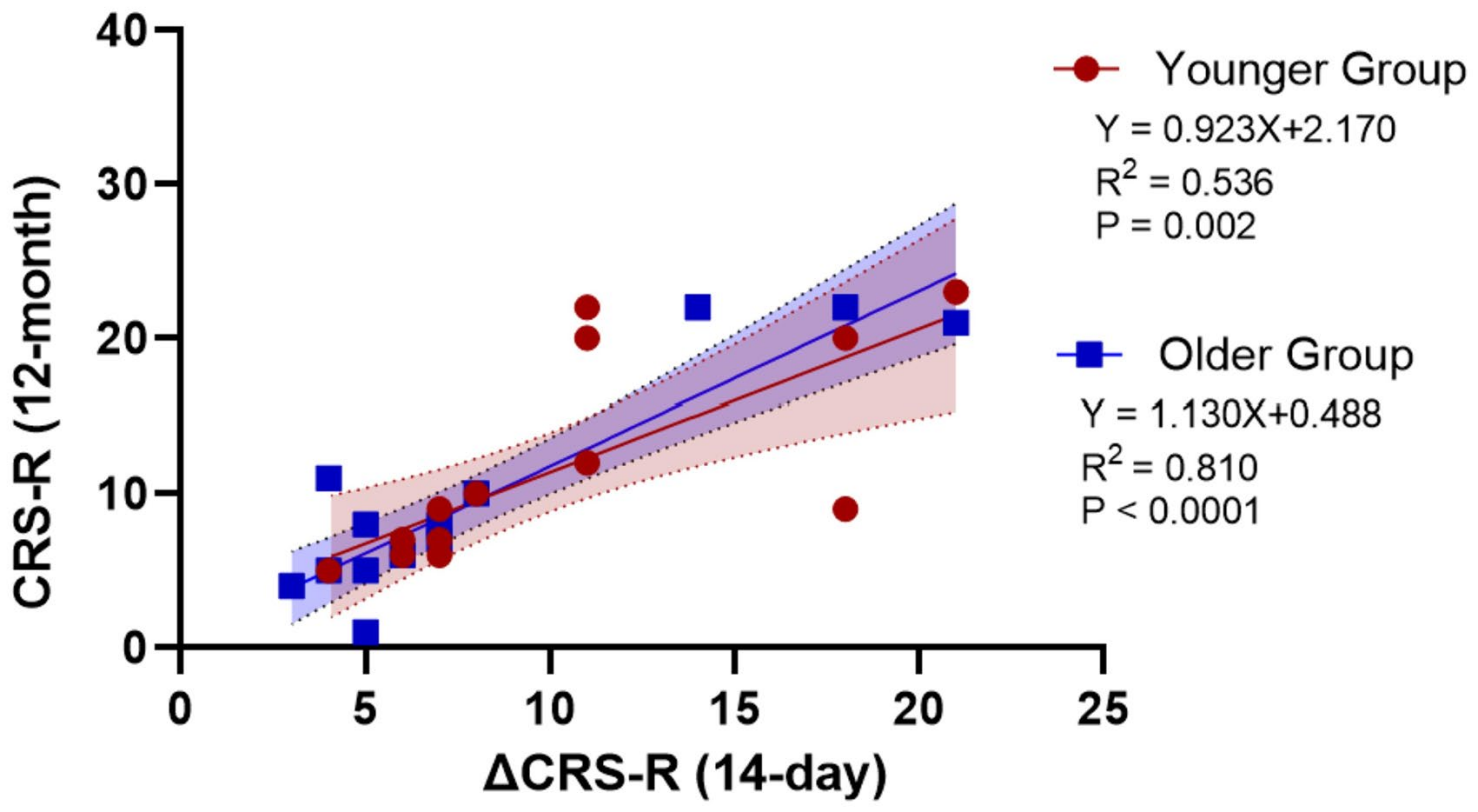
Association between early neurological improvement and long-term recovery. Linear regression analysis showing the relationship between the 14-day ΔCRS-R and the CRS-R score at 12 months in the younger (red) and older (blue) groups. Solid lines represent fitted regression lines and shaded areas indicate the 95% confidence intervals.

To further evaluate whether early neurological improvement was associated with long-term functional recovery, separate linear regression analyses were performed in the younger and older groups (Figure 5). In the younger group, the 14-day ΔCRS-R was positively associated with the CRS-R score at 12 months (slope = 0.923, 95% CI 0.409–1.436, R^2^ = 0.536, *p* = 0.0019). A similar association was observed in the older group, with an even stronger goodness of fit (slope = 1.130, 95% CI 0.802–1.458, R^2^ = 0.810, *p* < 0.0001). These findings indicate that patients showing greater early neurological improvement tended to achieve higher CRS-R scores at 12 months in both age groups.

## 4 Discussion

This study showed that patients with prolonged disorders of consciousness experienced sustained improvements in CRS-R scores during the 24-month follow-up after stSCS. Because of the retrospective design and the absence of a control group, these findings indicate an association between stSCS and long-term neurological improvement rather than a direct causal effect. Early postoperative improvement was significantly associated with long-term CRS-R outcomes in both age groups. Collectively, these findings suggest that stSCS may provide durable clinical benefits in selected patients with pDoC and support further evaluation in prospective controlled studies.

The persistence of clinical benefits observed up to twenty-four months in this study provides important evidence regarding the long-term outcomes of stSCS. Previous investigations have predominantly evaluated the outcomes of spinal cord stimulation within shorter follow-up intervals ranging from several weeks to three months (6–10). Following 14 days of stSCS, over 70% of the patients demonstrated a meaningful clinical response, while 50% achieved a definitive diagnostic improvement in their consciousness state (6). In our previous study, a clinical improvement of 2.3 points was achieved at the end of the 14-day intervention and remained stable through the 3-month follow-up (4). While those studies demonstrated immediate neurofunctional improvements during or shortly after the stimulation phase, whether such short-term interventions could induce sustained clinical recovery remained an unresolved question in neuromodulation research. In the present cohort, improvements in CRS-R scores were maintained throughout the 24-month follow-up despite cessation of stimulation after 14 days.

Several previous studies have reported favorable outcomes following SCS in patients with pDoC, although considerable heterogeneity exists across patient populations and stimulation protocols (10, 12, 18). A recent systematic review involving 508 patients reported an overall effectiveness rate of 37% for SCS, with higher response rates in MCS (63%) than in UWS/VS (30%) (21). In our cohort, five patients (16.7%) reached EMCS within 14 days after stimulation. Although this early improvement appears encouraging, differences in patient selection, outcome measures, and follow-up duration preclude direct comparison with previous studies. Therefore, the observed findings should be interpreted cautiously and require validation in prospective controlled studies.

The sustained improvements observed during the 24-month follow-up suggest that the stSCS intervention may be associated with neurological changes that persist beyond the stimulation period. However, because this retrospective study did not include a control group or longitudinal neurophysiological assessments, the present findings demonstrate an association rather than a direct causal effect. One possible explanation is that short-term stimulation modulates the ascending reticular activating system and its widespread projections to the thalamus and cerebral cortex, thereby facilitating the recovery of large-scale consciousness networks (22, 23). Previous experimental and clinical studies have further suggested that SCS may induce activity-dependent neuroplastic changes that persist after cessation of stimulation (14). We hypothesize that the sustained behavioral improvements observed in this cohort may be related to activity-dependent neuroplasticity initiated during the stimulation period. Previous studies have proposed that exogenous electrical stimulation may alter neurotransmitter release (24, 25) and functional connectivity within large-scale brain networks (26). In particular, Sampaio-Baptista et al. demonstrated that a brief period of increased neural activity was sufficient to induce structural changes in interconnected white matter pathways within 24 h (13). Similarly, high-density EEG studies have shown that although stSCS may not immediately produce substantial behavioral improvement, widespread enhancement of frontoparietal and thalamocortical functional connectivity can already be detected during the stimulation period (27). Whether similar electrophysiological or structural changes underlie the long-term behavioral improvements observed in patients with pDoC following stSCS remains to be determined and warrants further investigation using longitudinal EEG and multimodal neuroimaging.

The present study suggests that age may influence the pattern of neurological recovery following stSCS. Younger patients demonstrated earlier within-group improvements in CRS-R scores and numerically greater ΔCRS-R values during follow-up, whereas longitudinal mixed-effects analysis showed no significant age-by-time interaction, indicating that the overall recovery trajectories were broadly comparable between age groups. These findings suggest that younger patients may exhibit earlier responsiveness to neuromodulation, while older patients retain the potential for continued functional recovery during long-term follow-up.

The descriptive differences in the timing of recovery observed between the two age groups might be explained by age-related differences in neuroplastic potential and physiological reserve (15, 28). Younger individuals generally exhibit greater structural and functional adaptability following brain injury, including enhanced axonal sprouting and synaptic remodeling (29–31), whereas aging is frequently associated with reduced repair capacity, microvascular alterations, and progressive white matter degeneration (32–34). However, because not much neurophysiological or neuroimaging data were collected in the present study, these mechanisms remain speculative and should be interpreted as biological hypotheses rather than direct evidence.

Despite differences in the timing of recovery, improvements in consciousness were maintained in both age groups throughout the 24-month follow-up. Although younger patients appeared to show greater early improvement, intergroup comparisons of ΔCRS-R at individual follow-up time points were not statistically significant after multiple-comparison correction, and no significant differences were observed in the distribution of consciousness states between the two groups. Considering that long-term outcomes may also be influenced by rehabilitation intensity, post-discharge medical management, systemic complications, and spontaneous recovery, age should not be regarded as an absolute criterion when considering patients for stSCS. Instead, appropriate patient selection should integrate multiple clinical factors rather than chronological age alone.

The present study further demonstrated that early neurological improvement was positively associated with long-term functional outcome. Patients who exhibited greater improvement in CRS-R scores at 14 days generally achieved higher CRS-R scores at 12 months in both age groups. Because the long-term outcome was evaluated using the absolute CRS-R score rather than the change from baseline, this analysis reduced the potential influence of mathematical coupling between variables and provides additional support for the clinical relevance of early behavioral responsiveness.

Although this association should not be interpreted as evidence of causality, it suggests that the neurological response observed during the short postoperative stimulation period may serve as a practical indicator of subsequent recovery. From a clinical perspective, assessment at 14 days may help identify patients who are more likely to achieve favorable long-term outcomes and may facilitate individualized rehabilitation planning and communication with caregivers. Future prospective studies incorporating multivariable prediction models and external validation cohorts are needed to determine whether early CRS-R improvement can be established as an independent potential prognostic indicator.

Several limitations of this study should be acknowledged. First, this was a retrospective single-center study with a relatively small sample size (n = 30), which may limit the generalizability of the findings. Second, the absence of a control or sham stimulation group precludes distinguishing the specific effects of stSCS from spontaneous recovery or other factors associated with the natural course of pDoC. Third, although all patients received standardized treatment during hospitalization, rehabilitation programs and concomitant medical management after discharge could not be fully standardized or controlled, which may have influenced long-term outcomes. Finally, neurological recovery was evaluated primarily using the CRS-R, and the lack of longitudinal neuroimaging or electrophysiological assessments limited further investigation of the neural mechanisms underlying the observed behavioral changes. Finally, deaths during follow-up resulted in missing longitudinal observations, although linear mixed-effects models were used to reduce the impact of incomplete repeated measurements. Larger prospective, multicenter controlled studies incorporating multimodal assessments are needed to further evaluate the efficacy and mechanisms of stSCS.

## 5 Conclusion

Patients with pDoC showed sustained CRS-R improvement after stSCS during long-term follow-up. Age appeared to influence the pattern of recovery, with younger patients showing earlier improvement, although overall long-term outcomes were comparable between age groups. Early improvement was associated with better long-term outcomes, suggesting potential prognostic value. The specific contribution of stSCS requires further validation in prospective controlled studies.

## Data Availability

The raw data supporting the conclusions of this article will be made available by the authors, without undue reservation.
